# Hyposalivation and Poor Dental Health Status Are Potential Correlates of Age-Related Cognitive Decline in Late Midlife in Danish Men

**DOI:** 10.3389/fnagi.2018.00010

**Published:** 2018-01-30

**Authors:** Christiane E. Sørensen, Naja L. Hansen, Erik L. Mortensen, Martin Lauritzen, Merete Osler, Anne M. L. Pedersen

**Affiliations:** ^1^Section of Oral Medicine, Clinical Oral Physiology, Oral Pathology and Anatomy, Department of Odontology, Faculty of Health and Medical Sciences, University of Copenhagen, Copenhagen, Denmark; ^2^Center for Healthy Aging, Faculty of Health and Medical Sciences, University of Copenhagen, Copenhagen, Denmark; ^3^Functional Imaging Unit, Diagnostic Department, Rigshospitalet-Glostrup, Glostrup, Denmark; ^4^Department of Public Health, Faculty of Health and Medical Sciences, University of Copenhagen, Copenhagen, Denmark; ^5^Danish Aging Research Center, Universities of Aarhus, Southern Denmark and Copenhagen, Odense, Denmark; ^6^Department of Neuroscience and Pharmacology, Faculty of Health and Medical Sciences, University of Copenhagen, Copenhagen, Denmark; ^7^Department of Clinical Neurophysiology, Rigshospitalet-Glostrup, Copenhagen, Denmark; ^8^Research Center for Prevention and Health, Rigshospitalet-Glostrup, Copenhagen, Denmark

**Keywords:** salivary secretion, xerostomia, DMFs index, oral health, age-related changes in cognition, central autonomic control

## Abstract

**Introduction:** Peripheral correlates of age-associated cognitive decline are important tools in the screening for potentially abnormal courses of cognitive aging. Since salivary gland function is controlled by the autonomic and central nervous system, associations between cognitive changes and salivary gland hypofunction were tested in two groups of middle-aged men in late midlife, who differed substantially with respect to their midlife performance in verbal intelligence when compared with their performance in young adulthood.

**Materials and Methods:** Participants (*n* = 193) were recruited from the Danish Metropolit Cohort of men born in 1953. Based on their individual change in performance in two previously administered intelligence tests, they were allocated to one group of positive and one group of negative outliers in midlife cognition scores, indicating no decline versus decline in test performance. All participants underwent a clinical oral examination including assessments of their dental, periodontal, and mucosal conditions. Whole and parotid saliva flow rates were measured, and the number of systemic diseases and medication intake as well as daytime and nocturnal xerostomia were registered.

**Results:** Participants with decline in cognitive test performance in midlife had significantly lower unstimulated whole saliva flow rates, higher prevalence of hyposalivation and daytime xerostomia and a higher caries experience than participants with no decline in midlife performance. Daytime and nocturnal xerostomia were associated with daily intake of medication and alcohol.

**Discussion:** Overall, hyposalivation, xerostomia and poor dental status distinguished a group of men displaying relative decline in cognitive performance from a group of men without evidence of cognitive decline. Thus, hyposalivation and poor dental health status may represent potential correlates of age-related cognitive decline in late midlife, provided that other causes can be excluded.

## Introduction

Sensitive predictors of different courses of cognitive aging are important tools in the screening for persons at risk of transition into an age-associated cognitive disorder. As central nervous system predictors of subtle cognitive changes are difficult to identify, it is of interest to find clinical and peripheral correlates which can be assessed more easily.

Salivary gland function is under autonomic control and also regulated by higher order brain centers, many of which participate in a central autonomic network of connected brain structures at forebrain, midbrain and brainstem levels (Pedersen et al., 2012). Thus, it is well-known that for example mental stimuli or affective states can influence saliva secretion, and the relation between cognitive processes and saliva secretion has found use in psychophysiological studies. The close relationship between the central and the autonomic nervous system indicates that central autonomic control pathways may be affected by degenerative processes that also underlie cognitive deterioration. Changes of autonomic functions have been observed in patients with Alzheimer’s disease (Aharon-Peretz et al., 1992; Algotsson et al., 1995), and it has also been shown that untreated patients with Parkinson’s disease (Cersósimo et al., 2009) and Alzheimer’s disease (Ship et al., 1990; Ship and Puckett, 1994) have lower saliva flow rates than healthy persons. Previous studies on the potential relation between cognitive decline and autonomic dysfunction in persons with cognitive disorders suggest that functional aspects of salivary glands might predict cognitive decline analogous to cardiovascular correlates that relate to impaired cognition (Kim et al., 2006; Collins et al., 2012). Hyposalivation, a condition characterized by abnormal low unstimulated and/or stimulated whole saliva flow rates (Sreebny, 2000), and hence also xerostomia (the sensation of dry mouth) constitute potential autonomic nervous system correlates of cognitive decline. Hyposalivation may reflect lowered parasympathetic activity as parasympathetic stimulation mediates the largest fluid output from the glands (Pedersen et al., 2012).

The objective of this study was to identify autonomic nervous system correlates of cognitive decline. We hypothesized that men displaying decline in cognitive performance in late midlife have lower saliva flow rates and poorer oral health than age-matched men with no evidence of decline in cognitive performance.

## Materials and Methods

### Study Participants

This study was part of a multidisciplinary project on predictors of cognitive changes in middle-aged men recruited from the Danish Metropolit Cohort of the Copenhagen Aging and Midlife Biobank (CAMB) project (Osler et al., 2006; Osler et al., 2013; Lund et al., 2015). For further details about the recruitment and selection procedures and previous published papers related to the project (Hansen et al., 2014; Sørensen et al., 2014, 2016, Waller et al., 2016). Briefly, all participants were born in 1953, and selected in 2009 on basis of their performance in two earlier intelligence tests, the Børge Priens Prøve (BPP) (Teasdale, 2009) at about age 18 and the Intelligence-Struktur-Test (I-S-T 2000 R) (Amthauer et al., 2001) at age 56 (Osler et al., 2013; Mortensen et al., 2014). The correlation between the two tests was found to be 0.70 with an almost 40-year retest interval (Osler et al., 2013). Based on the BPP test scores the expected I-S-T 2000 R scores in midlife were predicted by a regression model. The largest positive or negative standardized residuals between predicted and obtained I-S-T 2000 R scores were used to select a group with no evidence of cognitive decline (group 1) and a group with an indication of decline in cognitive performance in midlife (group 2). Exclusion criteria were known medical conditions such as neurodegenerative or major psychiatric disorders, dementia, major brain lesions, alcohol or drug abuse. A total number of 195 men participated, and 193 were included. All participants received verbal and written information about the examinations, and the project was approved by the ethical committee of the Capital Region of Denmark (no. H-3-2010-016). All participants gave written consent. The study was performed following the STROBE recommendations.

### Assessment of Xerostomia and Measurements of Saliva Flow Rates

All participants were interviewed using a standardized questionnaire in which current and previous diseases, medication intake, smoking, alcohol habits, and symptoms of oral dryness during morning and daytime (daytime xerostomia), and during night-time (nocturnal xerostomia) were registered. Daytime xerostomia was rated as described previously (Sørensen et al., 2014), and nocturnal xerostomia was assessed by questioning about any symptoms of oral dryness during night-time and/or frequent wake-ups feeling thirsty, coded into yes (1) or no (0). Measurements of unstimulated whole saliva (UWS), paraffin-chewing-stimulated whole saliva (SWS), and citric acid (2%) stimulated parotid saliva (SPS) flow rates were performed as earlier described in detail (Pedersen et al., 1999). UWS was sampled over a 10-min period, SWS and SPS over a 5-min period. Samples with values ≤0.10 ml/min for UWS flow rates were designated as hyposalivation (Pedersen et al., 1999). Since the frequency of hyposalivation for SWS flow (i.e., ≤0.70 ml/min) was below 5, it was not further considered for statistical analysis.

### Clinical Oral Examination

Any changes of the oral mucosa and presence of oral mucosal diseases/lesions were registered. Oral hygiene status and the degree of gingival inflammation were scored at four sites per tooth on six index teeth (16, 21, 24, 36, 41, and 44) using the plaque and gingival indices (Pedersen et al., 1999). The periodontal probing pocket depth was scored on the same index teeth to assess the presence of periodontitis. The number of decayed (D), missing (M) and filled (F) surfaces (s) (DMF-s) and teeth (t) (DMF-t) and the total number of these was expressed as the DMF-s and -t indices. For analyses we used the DMF-s index. Third molars were excluded in all calculations.

### Statistics

SAS 9.3 (SAS Institute Inc., Cary, NC, United States) and GraphPad Prism version 6, GraphPad Software, United States, were used for analyses. Significance of group differences for categorical variables was calculated by Chi-Square tests and by Fisher’s exact test when the number of observations was below five. The number of diseases and medications taken on a daily basis were coded into: 0 (= none) or 1 (= 1 or more within the same major category). Ordinal categorical and continuous data were analyzed with the Wilcoxon Two Sample Rank Sum Test, as not all variables were assumed to approximate a normal distribution, except for the BPP and IST-2000-R cognition scores, which were analyzed by an unpaired *t*-test. *P*-values < 0.05 were considered statistically significant. Associations between ordinal and/or continuous variables were tested by Spearman’s rank correlation coefficient and associations between categorical and continuous variables were tested by the Kruskal–Wallis Test. When multiple correlations were tested for the same variable, the alpha level of significance of 0.05 was adjusted by the Holm-Bonferroni method for each family of tests. Adjusted *P*-values ≥0.05 were not considered statistically significant. All tests were two-sided.

## Results

Demographic data and cognitive scores of the two groups have been reported previously (Hansen et al., 2014; Sørensen et al., 2014, 2016, Waller et al., 2016). Briefly, the mean (±SD) age was 57.86 ± 0.06 years in group 1 (*n* = 94) and 58.04 ± 0.08 years in group 2 (*n* = 99). The mean number of years of education differed from 14.06 ± 2.28 in group 1 to 12.65 ± 2.24 years in group 2 (*P* < 0.0001). No group difference was found with respect to the participant’s occupational status. Data were available from 176 men and 95.5% of them were employed (95.4% out of 87 in group 1 and 95.5% out of 89 in group 2). The two groups did not differ significantly with respect to smoking habits or alcohol consumption (Sørensen et al., 2016). The cognitive performance scores applied to the number of participants included in this study also resembled those shown in the previous report, i.e., the mean BPP score in early adulthood was 46.0 ± 9.7 for group 1, and 45.4 ± 8.2 for group 2 (*P* = 0.61), whereas the mean I-S-T 2000 R cognitive scores in late midlife varied from 42.6 ± 7.4 in group 1 to 21.1 ± 6.0 in group 2 (*P* < 0.0001). Occupational status, alcohol and smoking habits were not significantly correlated with the I-S-T 2000 R performance scores.

The most frequently reported medical conditions were musculoskeletal diseases (28.5%), hypertension (29.5%), allergies (22.8%), and hypercholesterolemia (22.3%) (**Table 1**). While the latter three categories were almost evenly distributed between the two groups, musculoskeletal diseases were about 1.9 times more prevalent in group 2 (*P* = 0.01), and gastrointestinal diseases about 3.3 times more prevalent in group 2 than in group 1 (*P* = 0.03). None of the disease categories was correlated with cognitive performance scores or saliva flow rates at adjusted significance levels.

**Table 1:** Distribution of diseases reported by the 193 male participants born in 1953.

ICD-10 classification system	Group 1 *n* = 94 (%)	Group 2 *n* = 99 (%)	All *n* = 193 (%)	*P*-value
Malignant neoplasms^a^	3.2	6.1	4.7	0.50
Disorders of the thyroid gland^b^	0	3	1.6	0.25
Diabetes mellitus^c^	4.3	3	3.6	0.72
Hypercholesterolemia^d^	20.2	24.2	22.3	0.50
Depressive episode^e^	10.6	7.1	8.8	0.38
Persistent mood disorder^f^	0	4	2.1	0.12
Migraine^g^	10.6	5.1	7.8	0.15
Essential hypertension^h^	30.9	28.3	29.5	0.70
Other cardiac diseases^i^	13.8	9.1	11.4	0.30
Allergic and atopic disorders^j^	23.4	22.2	22.8	0.84
Chronic lower respiratory diseases^k^	3.2	6.1	4.7	0.50
Diseases of the digestive system^l^	4.3	13.1	8.8	0.03^∗^
Allergies to medicaments^m^	3.2	4	3.6	0.99
Arthropathies^n^	4.3	8.1	6.2	0.27
Musculoskeletal diseases^o^	20.2	36.4	28.5	0.01^∗^
Diseases of the genitourinary system^p^	3.2	3	3.1	0.99
Psoriasis^q^	6.4	1	3.6	0.06

*List of reported disorders according to the International Classification of Diseases, ICD-10 system (WHO, 2016a): ^*a*^(C09, C14, C20, C43, C44, C62); ^*b*^(E03, E05); ^*c*^(E10, E11); ^*d*^(E78.0); ^*e*^(F32); ^*f*^(F34); ^*g*^(G43.0, G43.1); ^*h*^(I10); ^*i*^(I22, I49, Q24, Z95, I61, I82); ^*j*^(J30, L20, L23, T78.1, T78.3); ^*k*^(J42, J44.9, J45); ^*l*^(K21, K51, K80, K44, K29, K40); ^*m*^(reported allergy to drugs, medicaments and biological substances: Z88); ^*n*^(M06, M07, M10, D86); ^*o*^(M15, M48, M50, M51, M76.6, M77.1); ^*p*^(N20, N40); ^*q*^(L40). *P*-values obtained by Fisher’s exact test or Chi-Square test. ^∗^*P* < 0.05*.

Intake of lipid-modifying agents (16.6%), agents acting on the renin-angiotensin system (15%), calcium-channel blockers (13.5%) and antithrombotic agents (12.4%) was most often reported. Intake of neurological agents (*P* = 0.01) and antigout preparations (*P* = 0.03) was significantly higher in group 2 than in group 1 (**Table 2**). When tested at the adjusted significance levels, the intake of medication was neither significantly correlated with I-S-T 2000 R scores nor with saliva flow rates, except for the intake of diuretics (thiazides), which was significantly negatively correlated with SWS flow rates (*r*_s_ = -0.21, *P* = 0.003, and adjusted *P* = 0.046).

**Table 2:** Self-reported daily intake of prescribed medication by the 193 male participants born in 1953.

Medication categories according to the ATC classification system (code)	Group 1 *n* = 94 (%)	Group 2 *n* = 99 (%)	All *n* = 193 (%)	*P*-value
Proton pump inhibitors (A02BC)	4.3	9.1	6.7	0.18
Medication used in diabetes (A10)	3.2	3	3.1	0.99
Antithrombotic agents (B01A)	12.8	12.1	12.4	0.89
Diuretics, thiazides and potassium (C03AB01)	4.3	6.1	5.2	0.75
Beta blocking agents (C07)	5.3	6.1	5.7	0.82
Calcium channel blockers (C08)	9.6	17.2	13.5	0.12
Agents acting on the renin-angiotensin system (C09)	17	13.1	15	0.45
Lipid modifying agents (C10A)	18.1	15.2	16.6	0.58
Antiinflammatory and antirheumatic agents, NSAIDs (M01A)	3.2	5.1	4.1	0.72
Antigout preparations (M04A)	0	6.1	3.1	0.03^∗^
Analgesics (N02)	3.2	3	3.1	0.99
Agents acting on the nervous system (N03, N05 and N06)	0	7.1	3.6	0.01^∗^
Nasal preparations/antihistamines (R01, R06)	6.4	1	3.6	0.06
Drugs for obstructive airway diseases (R03)	3.2	5.1	4.1	0.72

*Medications are classified according to the Anatomical Therapeutic Chemical (ATC) Classification System (WHO, 2016b). NSAIDs = non-steroid anti-inflammatory drugs. *P*-values obtained by Fisher’s exact test or Chi-Square test. ^∗^*P* < 0.05*.

The majority of participants displayed saliva flow rates (ml/min) within normal ranges (**Table 3**). However, the median unstimulated whole saliva (UWS) flow rate in group 2 was 19.5% lower than in group 1 (*P* = 0.01), whereas there were no significant differences regarding the median chewing-stimulated (SWS) and citric-acid stimulated (SPS) whole saliva flow rates. The frequency of hyposalivation was six times higher in group 2 than in group 1 (*P* = 0.01) (**Table 4**). Additionally, the total number of low secretors, i.e., UWS flow rates <0.20 ml/min was higher in group 2 (*P* = 0.01). Daytime xerostomia was reported by 21.8% of all participants with a frequency in group 2 almost twice as high as in group 1 (*P* = 0.02). Nocturnal xerostomia was observed in 23.3% of all participants with a higher frequency in group 2, but not statistically significant. Analyses of the inter-relationship between single parameters of xerostomia showed that there was no significant correlation between daytime xerostomia and UWS flow rates, hyposalivation, or low secretion. Neither daytime xerostomia nor nocturnal xerostomia or hyposalivation were significantly associated with daily smoking. Testing the same parameters for correlation with daily alcohol consumption showed that this was neither significantly correlated to daytime xerostomia nor hyposalivation, but to nocturnal xerostomia (*r*_s_ = 0.19, *P* = 0.01, and adjusted *P* = 0.03).

**Table 3:** Median values (ml/min) of unstimulated whole saliva (UWS), chewing-stimulated whole saliva (SWS), and citric-acid stimulated parotid saliva (SPS) flow rates in group 1 and group 2.

	Group 1	Group 2	All	*P*-value
UWS, ml/min	0.41 (0.05–2.02) *n* = 94	0.33 (0.04–1.5) *n* = 98	0.36 (0.04–2.02) *n* = 192	0.01^∗^
SWS, ml/min	2.61 (0.84–7.99) *n* = 93	2.26 (0.29–7.47) *n* = 99	2.51 (0.29–7.99) *n* = 192	0.21
SPS, ml/min	0.51 (0.10–1.60) *n* = 94	0.46 (0.04–1.30) *n* = 97	0.5 (0.04–1.60) *n* = 191	0.10

*P-values for group differences were calculated by Wilcoxon Two Sample Rank Sum test. *n*: number of participants. ^∗^*P* < 0.05*.

**Table 4:** Prevalence of hyposalivation (UWS ≤ 0.10 ml/min), low secretion (UWS < 0.20 ml/min), daytime and nocturnal xerostomia in group 1 and 2 and in the whole study population.

	Group 1 (%)	Group 2 (%)	All (%)	*P*-value
Hyposalivation	2.1	12.2	7.3	0.01^∗^
Low secretion	13.8	28.6	21.4	0.01^∗^
Daytime xerostomia	14.9	28.3	21.8	0.02^∗^
Nocturnal xerostomia	18.1	28.3	23.3	0.09

*Analysis of differences between groups was performed by Chi-Square test. ^∗^*P* < 0.05*.

At adjusted significance levels, there were no associations between diseases, hyposalivation, low salivary secretion, daytime or nocturnal xerostomia. The potential xerogenic effect of medication was also investigated by testing correlations between intake of the different medications and saliva flow rates and xerostomia. At the adjusted significance levels, intake of diuretics (thiazides) was positively correlated to low secretion (*r*_s_ = 0.22, *P* = 0.002, and adjusted *P* = 0.03), and intake of proton pump inhibitors was positively correlated with daytime xerostomia (*r*_s_ = 0.22, *P* = 0.002, and adjusted *P* = 0.03).

Almost all participants attended their dentist on a regular basis. The groups differed with respect to the total number of DMF-s (*P* = 0.03) (**Table 5**). The mean score of DMF-s in group 2 was 11.6% higher than in group 1. Furthermore, the number of filled tooth surfaces tended to be higher in group 2. The DMF-s was significantly negatively correlated to the cognitive performance scores (*r*_s_ = –0.18, *P* = 0.01, adjusted *P* = 0.04).

**Table 5:** Dental and oral health measures in group 1 and 2 as well as in the whole study population.

Dental status	Group 1 *n* = 94	Group 2 *n* = 99	All *n* = 193	*P*-value
No. of DMFs	37.96 (22.04)	42.94 (19.26)	40.51 (20.76)	0.03^∗^
No. of decayed surfaces	0.21 (0.53)	0.54 (1.84)	0.38 (1.38)	0.51
No. of missing surfaces	6.33 (16.45)	6.16 (10.73)	6.24 (13.78)	0.71
No. of filled surfaces	31.41 (16.15)	36.09 (16.43)	33.81 (16.42)	0.05
Plaque index	0.65 (0.74)	0.81 (0.89)	0.73 (0.82)	0.20
Gingival index	0.54 (0.64)	0.6 (0.74)	0.57 (0.69)	0.62
Chronic periodontitis	12.8%	15.2%	14%	0.63

*DMF-s index: Number of decayed, missing, filled surfaces given as mean values and standard deviation (SD). Wilcoxon Two Sample Rank Sum test was used for calculation of *P*-values for group differences within DMF-s scores, and the plaque and gingival indices. Significance of difference within the prevalence of periodontitis was tested by Chi-Square test. *n*: number of participants included. ^∗^*P* < 0.05*.

The two groups did not differ significantly with respect to the plaque and gingival indices, or the presence of periodontitis (**Table 5**). Periodontitis was significantly positively associated with the GI (*r*_s_ = 0.32, *P* < 0.0001).

The most prevalent oral mucosal changes observed in all participants were fissured (18%) and geographic tongue (9%). Also, mandibular tori (11%) were common. There were no differences between groups with regard to the presence of these mucosal changes or of oral candidosis, which was seen in 4% of all participants.

## Discussion

We have shown that salivary gland function, dental health, and xerostomia distinguished two groups of middle-aged men, who differed significantly with respect to midlife cognitive performance. None of the participants had a clinical diagnosis of cognitive impairment or any neurodegenerative disorders.

Similar to other autonomic parameters, measures of salivary gland function can be confounded by systemic diseases, medication and health-compromising life-style habits. At adjusted significance levels, diseases, smoking and daily alcohol intake were not associated with hyposalivation, daytime xerostomia or low saliva flow rates. However, intake of thiazides remained significantly positively correlated with low secretion, i.e., UWS < 0.2 ml/min, and negatively related to SWS flow rates, which is in line with previous observations (Nederfors et al., 1989; Smidt et al., 2010). Moreover, intake of proton pump inhibitors was associated with daytime xerostomia. Previous results are conflicting regarding the impact of, for example, the proton pump inhibitor Omeprazole^®^ on salivary gland function (Teare et al., 1995; Namiot et al., 2001). Nocturnal xerostomia was associated with daily intake of alcohol indicating that nocturnal xerostomia itself has limited potential as correlate of age-related changes in cognition.

Previous studies have also reported an association between poor oral and dental health and low cognitive performance in middle-aged adults (Stewart et al., 2008) and older adults (Stewart and Hirani, 2007). We showed that the caries load was higher in men with lower cognitive performance in midlife, and that the DMF-s score was negatively correlated to midlife cognitive performance. Our findings are in line with those from a previous study on adults in earlier midlife (Sabbah and Sheiham, 2010), apart from the fact that almost all of the participants in our study, including those with low cognitive scores, visited the dentist on a regular basis, and caries lesions were treated as evidenced by the high number of filled surfaces in group 2. There were no differences in the plaque and gingival indices between groups indicating comparable oral hygiene habits in both groups. Consequently, the higher caries prevalence found in men with decline in cognitive performance is more likely related to salivary gland dysfunction than to poor oral hygiene, as saliva is essential for the maintenance of oral health. Hyposalivation was more common and the UWS flow rates lower in group 2 than in group 1. Parasympathetic nerve impulses induce the largest fluid secretion from the salivary glands (Pedersen et al., 2012), and salivary gland hypofunction that cannot be ascribed to medication intake, trauma or behavioral factors may be of neurogenic origin. We speculate that dysregulation of the central autonomic network, i.e., neuronal circuits that include limbic system or cortical structures involved in regulation of preganglionic parasympathetic activity, may lead to altered parasympathetic outflow and reduced saliva secretion. These speculations are supported by a study showing that the parasympathetic system was affected in persons with mild cognitive impairment and an association between impaired autonomic functions and poorer cognitive performance (Collins et al., 2012). On the other hand, the low frequency of hyposalivation regarding stimulated whole saliva indicates that the ability of the salivary glands to respond adequately to masticatory and gustatory stimuli was unaltered in both groups. Accordingly, parasympathetic mechanisms related to the formation of unstimulated saliva could be more prone to be affected by central changes. The preganglionic parasympathetic innervation of the minor mucous and sublingual salivary glands as well as the submandibular glands, which contribute most to unstimulated saliva, originates from the superior salivatory nucleus in the brainstem. Studies on the rodent brain have shown that cortical and subcortical structures, which engage in central autonomic control, project to the superior salivatory nucleus (Jansen et al., 1992; Hübschle et al., 1998). These identified structures included telencephalic components such as the insular cortex (Hübschle et al., 1998), the bed nucleus of the stria terminalis and the central nucleus of the amygdala (Jansen et al., 1992; Hübschle et al., 1998). Together with regions of the medial prefrontal cortex, they engage “in high-order processing of viscerosensory information and initiation of integrated autonomic responses” (Benarroch, 1993). These observations may provide a neuroanatomical substrate for a relation between higher cognitive and affective processes and salivary gland function, which in turn could be reflected by changes in salivary flow and composition in association with altered cognitive or affective states.

The present study has the strength that it is longitudinal in its design and includes a well-characterized, large cohort of same age and gender. While the latter circumvents confounding by these two factors, it implicates on the other hand that further research is necessary to test the generalizability of our findings in a study group representative of a broader population.

## Conclusion

Hyposalivation, xerostomia and poor dental status distinguished a group of men displaying relative decline in cognitive performance from a group of men without evidence of cognitive decline. These findings could not be explained by age *per se*, since all participants had the same age, nor unhealthy behavior such as smoking, alcohol consumption or avoidance of regular dental check-ups. Neither is it likely that disease or medication alone is responsible for the group differences. Our findings suggest a linkage between altered central autonomic control pathways and altered central pathways involved in cognitive processing.

## Author Contributions

Substantial contribution to the conception and design of the research, analyses, and interpretation of the data: CS, NH, EM, ML, MO, and AP. Drafted the manuscript: CS. All authors have read and critically revised the manuscript for its intellectual content and approved the final version.

## Conflict of Interest Statement

The authors declare that the research was conducted in the absence of any commercial or financial relationships that could be construed as a potential conflict of interest.
